# Hemodynamic effects of intravenous paracetamol in critically ill children with septic shock on inotropic support

**DOI:** 10.1186/s40560-020-0430-0

**Published:** 2020-01-29

**Authors:** Elhanan Nahum, Avichai Weissbach, Eytan Kaplan, Gili Kadmon

**Affiliations:** 1 Pediatric Intensive Care Unit, Schneider Children’s Medical Center of Israel, 14 Kaplan St., 4920235 Petach Tikva, Israel; 2 Sackler Faculty of Medicine, Tel Aviv University, Tel Aviv, Israel

**Keywords:** Paracetamol, Hypotension, Septic shock, Inotropic, Children

## Abstract

**Background:**

Treatment with intravenous paracetamol may impair hemodynamics in critically ill adults. Few data are available in children. The aim of this study was to investigate the frequency, extent, and risk factors of hypotension following intravenous paracetamol administration in children with septic shock on inotropic support.

**Methods:**

We retrospectively reviewed the electronic medical charts of all children aged 1 month to 18 years with septic shock who were treated with intravenous paracetamol while on inotropic support at the critical care unit of a tertiary pediatric medical center in 2013–2018. Data were collected on patient demographics, underlying disease, Pediatric Logistic Organ Dysfunction (PELOD) score, hemodynamic parameters before and up to 120 min after paracetamol administration, and need for inotropic support or intravenous fluid bolus. The main outcome measures were a change in blood pressure, hypotension, and hypotension requiring intervention.

**Results:**

The cohort included 45 children of mean age 8.9 ± 5.1 years. The mean inotropic support score was 12.1 ± 9.5. A total of 105 doses of paracetamol were administered. The lowest mean systolic pressure (108 ± 15 mmHg) was recorded at 60 min (*p* = 0.002). Systolic blood pressure decreased at 30, 60, 90, and 120 min after delivery of 50, 67, 61, and 59 drug doses, respectively. There were 5 events of systolic hypotension (decrease of 1 to 16 mmHg below systolic blood pressure hypotensive value). Mean arterial pressure decreased by ≥ 15% in 8 drug doses at 30 min (7.6%, mean − 19 ± 4 mmHg), 18 doses at 60 min (17.1%, mean − 20 ± 7 mmHg), 16 doses at 90 min (15.2%, mean − 20 ± 5 mmHg), and 17 doses at 120 min (16.2%, mean − 19 ± 5 mmHg). Mean arterial hypotension occurred at the respective time points in 2, 13, 10, and 9 drug doses. After 12 drug doses (11.4%), patients required an inotropic dose increment or fluid bolus.

**Conclusions:**

Hypotensive events are not uncommon in critically ill children on inotropic support treated with intravenous paracetamol, and physicians should be alert to their occurrence and the need for intervention.

## Background

Paracetamol was introduced in the late nineteenth century and is currently one of the most common antipyretic, analgesic drugs used worldwide. The mechanism of action of paracetamol is unclear and may involve inhibition of the cyclooxygenase, cannabinoid, or nitric oxide pathways in the central nervous system [1]. Perfalgan™ (acetaminophen) is an intravenous formulation of paracetamol developed in 2003 for use in critically ill patients. According to the company’s product information leaflet [2], the rate of hypotension complicating intravenous paracetamol treatment ranges from 0.01 to 0.1%. However, recent studies in critically ill adults reported a much higher incidence [3–9]. Cantais et al. [3] observed a 15% reduction in mean arterial pressure (MAP) in 52% of critically ill adults receiving intravenous paracetamol; in 35% of them, intervention in the form of a fluid bolus or an increase in vasopressor dose was required.

Complementary data in the pediatric medical literature are sparse. A case report by Yaman et al. [10] described severe hypotension and cardiac arrest in a child treated with intravenous paracetamol. Allegaert and Naulaers [11], in a study of 72 paracetamol-treated neonates, found that 8, all of whom had relatively low blood pressure already before treatment, experienced hypotension after treatment. The authors concluded that impaired hemodynamics in a neonate is a relative contraindication for intravenous paracetamol. Others, using pulse contour analysis, reported a significant, 4.7% reduction in MAP in critically ill children after paracetamol administration which they attributed to systemic vascular resistance [12]. Similarly, a study in children after cardiac surgery found that 1 in every 20 administrations of paracetamol (5%) was associated with a 15% drop in MAP from baseline [13]. However, this study, like the previous one [12], evaluated the change in MAP, not true hypotension.

The aim of the present study was to evaluate the frequency, extent, and risk factors of hypotension associated with intravenous administration of paracetamol in children with septic shock supported with inotropic drugs.

## Methods

A retrospective chart review study design was used. The study was approved by the institutional review board. The cohort included all children hospitalized in the pediatric intensive care unit (PICU) of a tertiary pediatric medical center in 2013–2018 with septic shock [14] who were treated with intravenous paracetamol (acetaminophen; Fresenius Kabi Austria GmbH, Graz, Austria) at the time of inotropic support. The paracetamol was infused over 15 min at a dose of 7.5 mg/kg for infants (age ≤ 1 year) and 15 mg/kg (up to 1 g) for older children. Exclusion criteria were vasopressor inotropic score (VIS) < 5 [15] at the time of paracetamol administration; hemodynamic instability during the 4 h prior to paracetamol administration; treatment with any antihypertensive medication, either orally or intravenously; and absence of an arterial line during the 2 h before and after paracetamol administration. Hemodynamic instability was defined as systolic blood pressure below the age-adjusted hypotension value or need for increased inotropic support to maintain systolic blood pressure above hypotension level during the 2 h prior to paracetamol administration [16]. Patients receiving oral paracetamol were excluded because its absorption was uncertain.

All patient parameters at our center are continuously and automatically recorded in the individual electronic charts (Metavision, *iMD*soft, Tel Aviv, Israel). For the present study, the following data were collected from the medical charts: patient age and sex; underlying disease; amount of sedative drugs; Pediatric Logistic Organ Dysfunction (PELOD) score; hemodynamic parameters (heart rate, systolic and diastolic blood pressure, MAP) immediately before paracetamol administration and 30, 60, 90, and 120 min after; and need for inotropic support or intravenous fluid bolus during the 4 h before and 2 h after paracetamol administration. The main outcome measures of the study were change in blood pressure, hypotension, and hypotension requiring intervention.

For each patient, we compared the hemodynamic findings after paracetamol administration to the pre-administration value. Below normal systolic, diastolic, and mean blood pressures were defined as blood pressure below the lower normal blood pressure limit, adjusted for age, according to the Pediatric Advanced Life Support (PALS) vital signs chart [17]. As for the PALS guidelines, systolic hypotension value may be lower than the lower normal blood pressure limit and defined as systolic blood pressure < 60 mmHg for neonates, < 70 mmHg for infants 1–12 months, and < 70 + (2xage in years) mmHg for children 1–10 years [17].

In addition, we calculated the differences between the systolic and diastolic blood pressure values and the respective normal means for age expressed as delta (Δ). A negative change of ≥ 15% in MAP after intravenous paracetamol administration was also evaluated, similar to previous studies [3, 9]. Temperature was measured either by rectal probe or temperature-sensing indwelling urinary catheters just before and 1 h after paracetamol administration.

Statistical analysis was performed with ©2018 QuickCalcs software (GraphPad software, San Diego, CA, USA). Changes in blood pressures, heart rates, and VIS were analyzed using analysis of variance (ANOVA) with repeated measures. Potential baseline factors predicting a risk of hypotension after paracetamol treatment were tested by ANOVA. For all tests, a *p* value of < 0.05 was considered statistically significant.

## Results

The cohort included 45 children who received a total of 105 intravenous paracetamol prescriptions (3 prescriptions were included in our earlier study of paracetamol [18]). Their demographic and baseline characteristics are shown in Table 1. There were 24 male and 21 female patients of mean age 8.9 ± 5.1 years (IQR 4.5–9.9 years) and mean weight 32.5 ± 21.0 kg (IQR 17–50 kg). Inotropic support was provided by various combinations of adrenaline, noradrenaline, dopamine, dobutamine, milrinone, and vasopressin. The mean VIS was 12.1 ± 9.5 (IQR 5–15.5).

**Table 1:** Demographic and clinical data of inotropic-supported children with septic shock treated with intravenous paracetamol

Characteristics	Value
Sex (M:F)	24:21
Age (years), mean ± SD (IQR)	8.9 ± 5.1 (4.9–13)
Weight (kg), mean ± SD (IQR)	32.5 ± 21.0 (17–50)
PICU patient category	
Surgical	3
Medical	14
Oncological	28
PELOD-2 score, mean ± SD	9.1 ± 2.1
Origin/background of septic shock	
Fever and neutropenia	12
Abdominal sepsis	8
Pneumonia	8
Urinary tract infection	4
Skin and deep tissue infection	4
Other	9
Causative organism	
Gram-negative	22
Gram-positive	8
Fungal	4
Negative blood culture	11
Vasopressor inotropic score, mean ± SD	12.1 ± 9.5
Number of paracetamol prescriptions	105
Indication for intravenous paracetamol*	
Fever (mean 38.6 ± 0.6 °C)	83
Pain	22
Mechanical ventilation	
Yes	83
No	22
Survived to PICU discharge	
Yes	42
No	3

Values given as *n* unless otherwise indicated

The mean dose of intravenous paracetamol was 12 ± 3 mg/kg. The indications for treatment were fever in 83 (79%) prescriptions and pain in 22. Paracetamol treatment led to a decrease in mean temperature from 38.6 ± 0.6 to 37.5 ± 0.8 °C (*p* < 0.001). In 83 cases, patients were being mechanically ventilated at the time paracetamol was prescribed. Three children did not survive to pediatric intensive care unit discharge.

### Hemodynamic effects of paracetamol—all patients/doses

#### Mean arterial blood pressure

For the whole cohort, the mean value of MAP before paracetamol administration was 79 ± 13 mmHg, and the lowest MAP value recorded after paracetamol administration was 77 ± 13 mmHg at 60 min. The difference between these values was not statistically significant (Table 2).

**Table 2:** Changes in hemodynamic parameters after intravenous paracetamol, whole cohort

Parameter	Time after paracetamol administration (no. of doses)					*p* value
	0 min (105)	30 min (105)	60 min (105)	90 min (105)	120 min (105)	
Mean systolic blood pressure (mmHg)	113 ± 15	112 ± 14	108 ± 15	109 ± 15	111 ± 16	*p* = 0.002
Mean diastolic blood pressure (mmHg)	62 ± 12	61 ± 12	60 ± 12	61 ± 13	62 ± 13	*p* = 0.25
Mean arterial blood pressure (mmHg)	79 ± 13	79 ± 13	77 ± 13	78 ± 13	79 ± 13	*p* = 0.14
Mean heart rate (bpm)	130 ± 27	130 ± 25	124 ± 27	122 ± 26	120 ± 26	*p* = 0.001
VIS	12.1 ± 9.5	12.2 ± 9.5	12.1 ± 8.9	12.4 ± 9.3	12.1 ± 8.7	*p* = 0.53
Mean Δ from lower limit of normal systolic pressure (mmHg)	13 ± 29	12 ± 14	8 ± 17	9 ± 17	11 ± 16	
Mean Δ from lower limit of normal diastolic pressure (mmHg)	6 ± 14	5 ± 14	4 ± 14	4 ± 16	5 ± 15	

All values are mean ± SD

*VIS* vasopressor-inotropic score, *Δ* delta

#### Systolic and diastolic blood pressures

For the whole cohort, mean systolic blood pressure before paracetamol administration was 113 ± 15 mmHg, and the lowest value after paracetamol administration was 108 ± 15 mmHg at 60 min. The difference between these values was statistically significant (*p* = 0.002). The lowest systolic blood pressure value was 8 ± 17 mmHg higher than the lower limit of normal systolic blood pressure, adjusted for age. There was no statistically significant change in diastolic blood pressure (Table 2). The mean heart rate at 90 and 120 min after paracetamol administration was significantly lower (*p* < 0.001) than the pre-paracetamol value.

#### Vasopressor inotropic score

There was no statistically significant change in VIS before and after paracetamol administration (Table 2).

### Hemodynamic effects of paracetamol—patients/doses with negative changes only

#### Mean arterial blood pressure

MAP decreased by ≥ 15% from the pre-paracetamol value in 8 doses (7.6%) at 30 min (mean drop − 19 ± 4 mmHg), 18 doses (17.1%) at 60 min (mean drop − 20 ± 7 mmHg), 16 doses (15.2%) at 90 min (mean drop − 20 ± 5 mmHg), and 17 doses (16.2%) at 120 min (mean drop − 19 ± 5 mmHg). Mean arterial hypotension occurred after 34 doses of paracetamol (32.4%); in 23 doses, hypotension was recorded at 60 and 90 min after paracetamol infusion (Table 3).

**Table 3:** Negative MAP changes, in patients with decrease in MAP, after intravenous paracetamol administration

Negative changes	Time after paracetamol administration			
	30 min	60 min	90 min	120 min
No. of events with ≥ 15% drop in MAP	8 (7.6%)	18 (17.1%)	16 (15.2%)	17 (16.2%)
Mean drop (mmHg)	− 19 ± 4	− 20 ± 7	− 20 ± 5	− 19 ± 5
No. of events with MAP below normal	2 (1.9%)	13 (12.4%)	10 (9.5%)	9 (8.6%)
Mean MAP below normal (mmHg)	− 6 ± 0	− 13 ± 4	− 13 ± 7	− 11 ± 4

*MAP* mean arterial pressure

#### Systolic blood pressure

Systolic blood pressure decreased from the pre-paracetamol value in 50 doses at 30 min, 67 doses at 60 min, 61 doses at 90 min, and 59 doses at 120 min. The maximal mean negative change was − 15 ± 12 mmHg, which occurred at 60 min. This value was still 2 ± 15 mmHg above the lower limit of normal systolic blood pressure and 16 ± 12 mmHg above the hypotensive value. Five children experienced systolic hypotension, with systolic blood pressure ranging from 1 to 16 mmHg below the lowest normal value (Table 4).

**Table 4:** Hemodynamics in patients with a decrease in systolic or diastolic blood pressure after intravenous paracetamol administration

Hemodynamic parameters	Time after intravenous administration (no. of doses)			
	30 min	60 min	90 min	120 min
	Negative change in systolic blood pressure			
	(50)	(67)	(61)	(59)
Mean drop in systolic pressure from *T*_0_ (mmHg)	− 12 ± 9 (− 7; − 14)	− 15 ± 12 (−5; − 22)	− 14 ± 13 (−5; − 21)	− 14 ± 12 (− 5; − 17)
Mean Δ from lower limit of normal systolic pressure (mmHg)	8 ± 14 (− 2; 15)	3 ± 14 (− 9; 14)	2 ± 15 (− 8; 14)	5 ± 14 (− 2; 15)
Mean Δ from systolic hypotension value (mmHg)	22 ± 12 (12; 29)	17 ± 13 (6; 26)	16 ± 12 (8; 26)	19 ± 13 (9; 26)
Number of patients with systolic hypotension	0	2 (− 1^a^, − 16^a^)	1 (− 1^a^)	2 (− 13^a^; − 5^a^)
	Negative change in diastolic blood pressure			
	*N* = 53	*N* = 59	*N* = 50	*N* = 52
Mean drop in diastolic pressure from *T*_0_ (mmHg)	− 7 ± 5 (− 11; − 3)	− 9 ± 7 (− 4; − 13)	− 9 ± 6 (− 4; − 13)	− 9 ± 7 (− 3; − 13)
Mean Δ from diastolic hypotension value (mmHg)	2 ± 12 (− 8; 11)	1 ± 14 (− 8; 12)	0 ± 14 (− 9; 9)	1 ± 13 (− 8; 9)

Values given as mean ± SD, (IQR) unless otherwise indicated

*Δ* delta, *T*_*0*_ time immediately before paracetamol transfusion

^a^mmHg below lowest normal systolic value for age

#### Diastolic blood pressure

Diastolic blood pressure decreased from the pre-paracetamol value in 53 doses at 30 min, 59 doses at 60 min, 50 doses at 90 min, and 52 doses at 120 min. The maximal mean drop in diastolic blood pressure occurred at 60, 90, and 120 min after paracetamol administration (Table 4).

#### Vasopressor inotropic score

There was no significant change in mean VIS among patients with a negative change in systolic blood pressure.

### Interventions

**Fig. 1:**
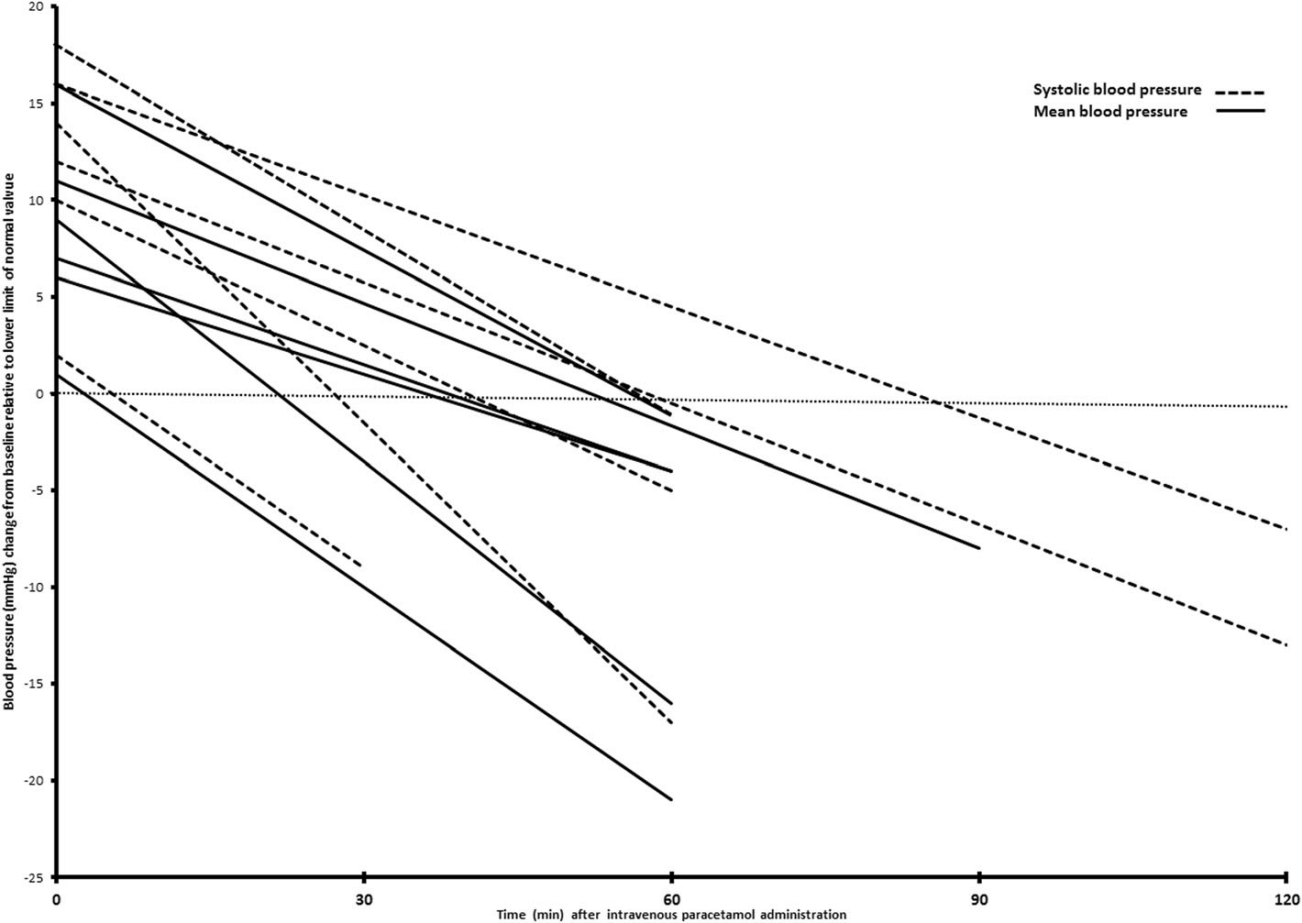
Systolic and mean arterial pressure changes from baseline, relative to lower limit of normal pressure, in patients required fluid bolus/VIS increment

Six patients required an increment in inotropic support (mean VIS increment 4.3 ± 1.7), and another 6 required fluid boluses to maintain systolic blood pressure or MAP within the normal range. Figure 1 describes the systolic and mean arterial pressure changes from baseline, relative to the lower limit of normal pressure, in these 12 patients before and after paracetamol infusion.

### Risk factors

There was no statistically significant association between baseline VIS and a decrease in MAP below the normal range (*p* = 0.49, ANOVA). Similarly, no statistically significant association was found between a drop in MAP to below normal and patient diagnosis (oncology/medical/surgical) (*p* = 0.52), amount of sedative drugs (morphine *p* = 0.28, midazolam *p* = 0.19), or indication (fever/pain) for paracetamol administration (*p* = 0.89). On stepwise logistic regression analysis, there was no association between these three variables (baseline VIS, patient diagnosis, indication) and a drop in MAP to below normal.

## Discussion

This study is the first to evaluate hypotension events after administration of intravenous paracetamol in children with septic shock on inotropic support. This patient group, which is highly hemodynamically vulnerable, often requires antipyretic medications. Thus, any hypotensive effect of paracetamol may be crucial. Our results showed that mean arterial hypotension was not an infrequent event after intravenous paracetamol administration, occurring after 34 doses (32.4%), and warranting intervention in 12 patients in the form of a fluid bolus or an increment in inotropic support. Among patients who experienced a decrease in systolic blood pressure after intravenous paracetamol, the highest number of negative events occurred at 60 min (67 events), with a decrease of − 15 ± 12 mmHg from the mean pre-paracetamol value.

The hypotensive effect of intravenous paracetamol is multifactorial and well recognized in the adult literature. More than 20 years ago, Brown [19] described two critically ill adults who experienced hypotension, unrelated to an allergic reaction, after receiving paracetamol. This finding was later supported by several studies in adults with and without a critical illness.

Kelly et al. [5] reported that of 196 intravenous or oral paracetamol administrations in 50 critically ill adults, 16 (8.2%) were associated with a hypotensive event, defined as a decrease of ≥ 20% in baseline systolic blood pressure or a systolic blood pressure of < 90 mmHg. In 11 of the 16 hypotensive episodes, there was a need for either a fluid bolus or increase in vasopressor infusion. Another study of 161 critically ill adults reported that 83 (51.9%) had hypotensive events, defined as a decrease of ≥ 15% in MAP, after intravenous paracetamol treatment, of whom 29 (35%) required therapeutic intervention [3]. Krajčová et al. [9] prospectively evaluated 6 critically ill adults treated with intravenous paracetamol on 45 occasions. In 45% of drug administrations, MAP dropped by ≥ 15%. Using pulse contour cardiac output analysis, the authors showed that the drop in MAP was due to a reduction in cardiac index and peripheral vasodilation and the consequent decrease in peripheral vascular resistance. Similar changes in skin blood flow and vascular resistance were reported by Boyle et al. [6].

In children with septic shock, two possible mechanisms may exaggerate the hypotensive effect of paracetamol. One is skin vasodilatation. Owing to the higher ratio of surface area to weight than in adults, skin vasodilatation occurring when temperature drops may exaggerate relative hypovolemia and facilitate hypotension. The other is the additive lowering effect of paracetamol on the already low cardiac output and myocardial depression characteristic of pediatric septic shock.

Ray et al. [12] reported a mean decrease of 4.8% (3.3 mmHg) in MAP from baseline (68 mmHg) in critically ill children. However, they included all routes of paracetamol administration, which may affect drug delivery and MAP change, and the reported decrease in MAP per se, which does not indicate true hypotension. Similarly, in pediatric patients with cardiac disease, Achuff et al. [13] defined hypotension as a negative change of 15% compared to baseline, and relative hypotension as a negative change of 10%. Although they did not report true hypotensive values, they concluded that 16% of the children experienced hypotension since they required fluid bolus or a vasoactive drug increment.

However, since MAP and heart rate would be expected to decrease to normal range on relief of fever and discomfort, the definition of hypotension as a decrease of > 15% mmHg in MAP can be misleading. Therefore, to identify true hypotension, we not only presented the negative changes in systolic and diastolic blood pressures and MAP after intravenous paracetamol, but also calculated the difference (Δ) in the post-paracetamol values from the lower normal range and hypotensive limit for age. We found that despite the drop in mean systolic blood pressure, the mean difference from the systolic hypotensive value was in the safe range, and only 5 events were by definition systolic hypotension. Furthermore, in only 34 of the 59 events in which MAP decreased by ≥ 15% relative to baseline, MAP was below the normal age-adjusted value. Thus, we suggest that rather than defining hypotensive events after paracetamol administration by the change from baseline, we should consider the change in blood pressure values (systolic/diastolic/mean arterial) relative to the normal range. Furthermore, since sedated children may experience low blood pressures, we suggest that low blood pressure together with the need for intervention is more clinically relevant than the change in pressure per se.

The present findings do not agree with our recently published study of 100 consecutive children treated with intravenous paracetamol [18], wherein no negative hemodynamic effect was found. The discrepancy is apparently due to the difference in the patient population. The cohort in the earlier study included all patients in the PICU of whom only 3 had septic shock. By contrast, the cohort in the present study was restricted to children with septic shock on inotropic support. It is possible that children on inotropic support are a unique critically ill patient subgroup requiring careful attention after intravenous paracetamol administration.

None of the potential risk factors evaluated in this study, i.e., baseline VIS, underlying diagnosis, sedative drugs, and indication for paracetamol administration, was associated with the development of hypotension after intravenous administration of paracetamol.

Our study has several limitations. First, although the data had been recorded electronically and continuously, they were collected retrospectively. Second, patients who received known hypotensive medications were excluded, but it is still possible that some patients experienced an unexpected hypotension event in response to some other medication that was given at the time of paracetamol administration. There are also other potential causes of hypotension in this setting, such as are unmet ongoing fluid loss and septic shock deterioration. Third, the cohort was relatively small, with some patients had more than one dose of paracetamol, and was not powered to identify all risk factors for hypotensive events after treatment.

## Conclusion

Clinicians should be alert to the occurrence of hypotensive events and the need for intervention following intravenous paracetamol administration in critically ill children with septic shock on inotropic support. Future prospective controlled studies are warranted to investigate below-normal blood pressures and the need to intervene and to further characterize the mechanisms and risk factors of paracetamol-induced hypotension in this patient group.

## Data Availability

All data were extracted from the patients’ electronic files and are available in the patient archives.

## Abbreviations

ANOVA: Analysis of variance
MAP: Mean arterial pressure
PELOD: Pediatric Logistic Organ Dysfunction
PICU: Pediatric intensive care unit
VIS: Vasopressor inotropic score
